# Supplementary figures and images for: Prolonged depletion of profilin 1 or F-actin causes an adaptive response in microtubules
Source: J Cell Biol. 2024 May 9;223(7):e202309097. doi: 10.1083/jcb.202309097 (PMC11082369; doi:10.1083/jcb.202309097)

Figure 2

2F

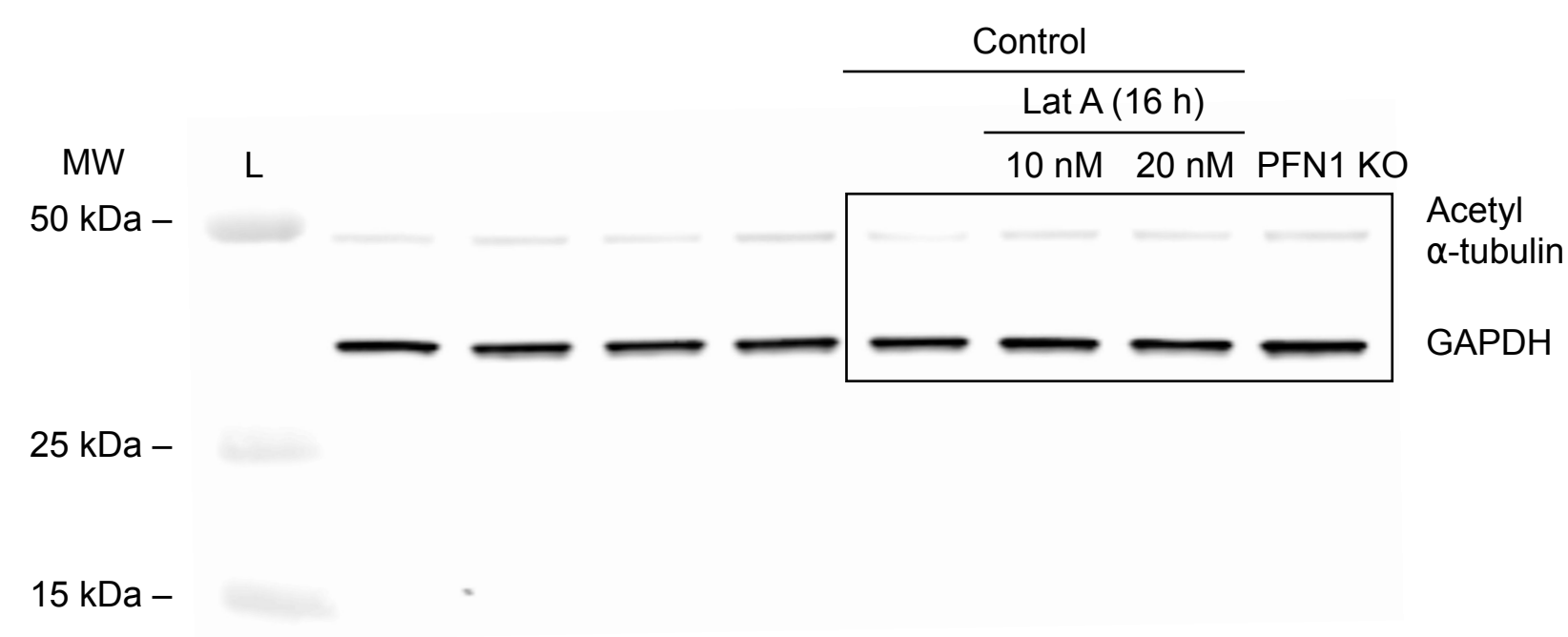

Supplement: SourceData F2 — is the source file for Fig. 2. [file JCB_202309097_SourceDataF2.pdf]

Figure 3

3H

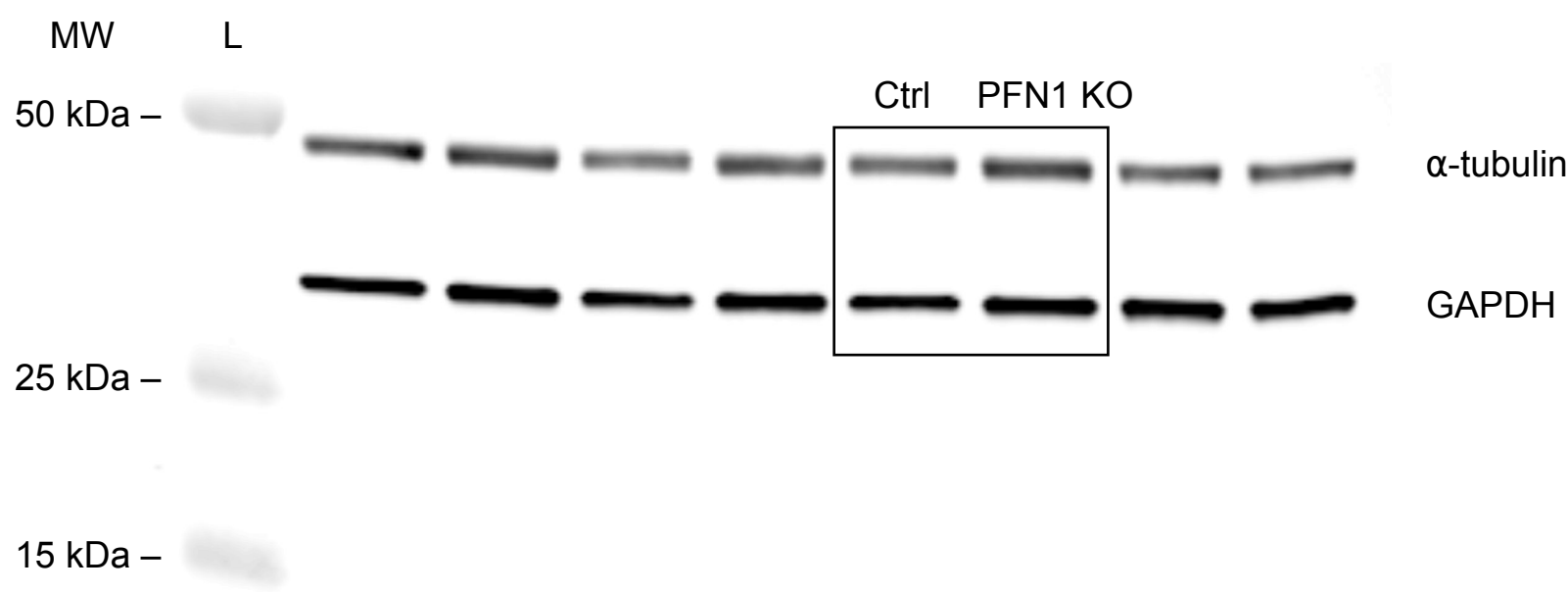

3I

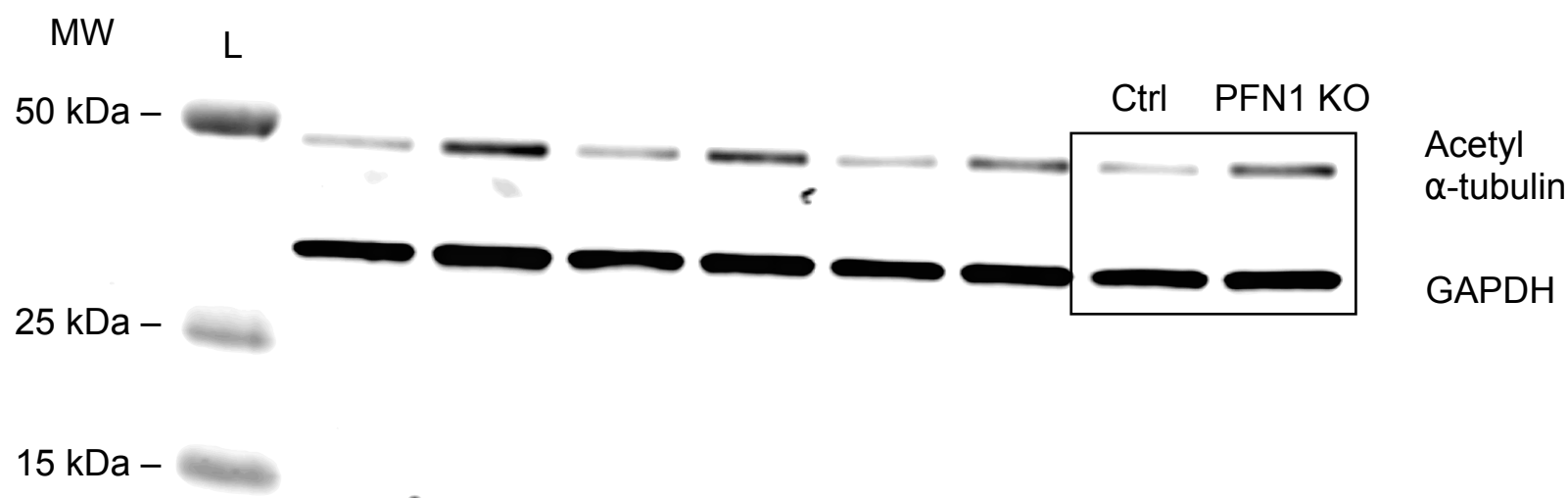

Supplement: SourceData F3 — is the source file for Fig. 3. [file JCB_202309097_SourceDataF3.pdf]

Supplementary Figure 3

S3B

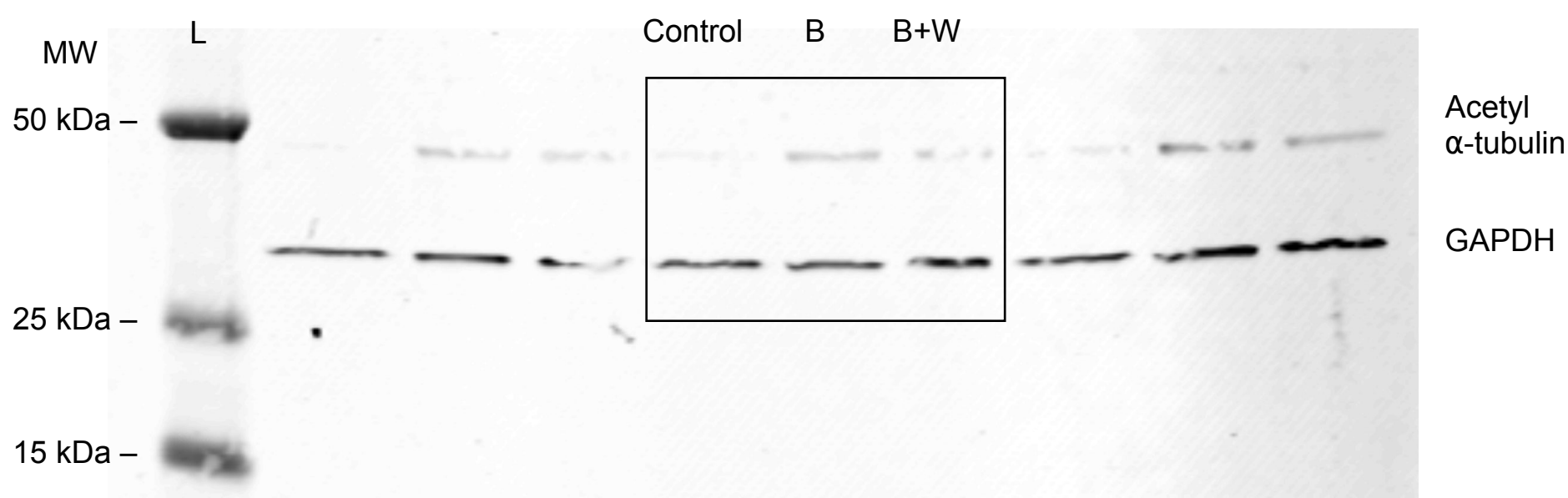

Supplement: SourceData FS3 — is the source file for Fig. S3. [file JCB_202309097_SourceDataFS3.pdf]
